# Validation of a Low-Cost Electromyography (EMG) System via a Commercial and Accurate EMG Device: Pilot Study

**DOI:** 10.3390/s19235214

**Published:** 2019-11-28

**Authors:** Sergio Fuentes del Toro, Yuyang Wei, Ester Olmeda, Lei Ren, Wei Guowu, Vicente Díaz

**Affiliations:** 1 Mechanical Engineering Department, Universidad Carlos III de Madrid, Avda. de la Universidad 30, 28911 Leganés, Spain; eolmeda@ing.uc3m.es (E.O.); vdiaz@ing.uc3m.es (V.D.); 2 Institute for Automotive Vehicle Safety (ISVA), Universidad Carlos III de Madrid, Avda. de la Universidad 30, 28911 Leganés, Spain; 3 School of Mechanical, Aerospace and Civil Engineering, University of Manchester, Manchester M13 9PL, UK; yuyang.wei@manchester.ac.uk (Y.W.); lei.ren@manchester.ac.uk (L.R.); 4 School of Science, Engineering and Environment, University of Salford, Salford M5 4WT, UK; G.Wei@salford.ac.uk

**Keywords:** low-cost sensors, electromyography, validation

## Abstract

Electromyography (EMG) devices are well-suited for measuring the behaviour of muscles during an exercise or a task, and are widely used in many different research areas. Their disadvantage is that commercial systems are expensive. We designed a low-cost EMG system with enough accuracy and reliability to be used in a wide range of possible ways. The present article focuses on the validation of the low-cost system we designed, which is compared with a commercially available, accurate device. The evaluation was done by means of a set of experiments, in which volunteers performed isometric and dynamic exercises while EMG signals from the rectus femoris muscle were registered by both the proposed low-cost system and a commercial system simultaneously. Analysis and assessment of three indicators to estimate the similarity between both signals were developed. These indicated a very good result, with spearman’s correlation averaging above 0.60, the energy ratio close to the 80% and the linear correlation coefficient approximating 100%. The agreement between both systems (custom and commercial) is excellent, although there are also some limitations, such as the delay of the signal (<1 s) and noise due to the hardware and assembly in the proposed system.

## 1. Introduction

Electromyography (EMG) provides information related to muscle activity [1,2,3]. For that reason, EMG devices are used in many research fields, such as biomedical, ergonomics, physiotherapy or sports performance applications, where it is very important to assess the behaviour of the muscles throughout the task [4] based on the changes in the electrical signal [5,6,7,8,9,10]. Moreover, this kind of technology can be used to improve other studies [11].

One of the main problems of existing EMG technology is the high cost of commercial devices. Some price examples of different commercial devices are shown in Table 1.

Although EMG signal acquisition can be done in different ways, nowadays one of the most used methods is by means of superficial electromyography (sEMG), because in comparison with other methods, e.g., needles, it is one of the less invasive methods. Some researchers [12] consider that sEMG is as valid as other methods, taking into account that the acquired signal must be denoised. Andrade et al. [12] offered various alternatives, explaining the positive and negative aspects to develop the filtering of the signal in their research, from a LPF (low-pass filter), wavelets, noise cancelation of the 50/60 Hz band or empirical mode decomposition (EMD).

Several researchers have tried to build or implement low-cost EMG systems. Supuk et al. [13] designed, developed and evaluated a low-cost EMG system, which can be used to measure the muscle activity during human motion, focused on the design of a cascade bio-amplifier that reduces the noise of the signal as a first step. Later they used different approaches to denoise the output signal. Validation was only centred on the gait analysis, where researchers measured the activity of six main muscles. Another example is the manuscript of Sophia Heywood et al. [14], where the authors compared the signal from a low-cost EMG system and a wire-commercial device. The evaluation consisted of the acquisition of the Vastus Lateralis signal while volunteers executed different exercises. After the authors denoised the signal using a few filters, they carried out an evaluation by means of the Teager–Kaiser energy operator (TKEO) and the maximal voluntary contraction (MVC) of the muscle, with good results. A third example is the work undertaken by Cheney et al. [15] where the authors developed the ability to adjust diverse variables, such as the gain, attenuation or offset. For that reason, they developed a 2-channel EMG board to gather a signal that later was filtered by low-pass filter. Unfortunately, the authors did not explain how the validation test was done.

Examples of applications of this technology cover a wide variety of fields, from the definition of the movement of a hand [16], up to the recognition of gestures inside a vehicle [17], in the field of rehabilitation [18,19,20], or even activity quantification during a deep brain stimulation intervention [21].

Up to the present, to our knowledge, there are no studies that compare the signal from a low-cost system and a wireless custom device, and identify limitations in the operation of the low-cost system. Thus, one of the first objectives of the present study is the comparison of different sEMG signals gathered from two systems: a low-cost system designed to be a user-friendly system [4] and a commercial system. Results of that analysis are intended to validate the low-cost sEMG system in order to examine whether the low-cost system was appropriate or if it needs improvements. For this purpose, an experiment was developed based on previous research by other authors who have used low-cost systems [13,14,16] or have tried to get a reliable signal from them [12,15].

In order to obtain both signals an experiment was designed in which diverse subjects executed isometric and dynamic exercises while EMG signals from the rectus femoris muscle (RF) were simultaneously gathered by a wireless commercial device (Delsys Trigno Wireless EMG System) and the designed low-cost system. Both signals were then analysed, and aspects of the comparison, including system limitations were explained.

Over the course of the study, the following hypotheses have been investigated:

**Hypothesis 1.**  *The low-cost system can provide a sEMG output signal that makes it possible to discern the exercise.*

**Hypothesis 2.**  *Based on previous studies [22] and related to muscle behaviour, the more difficult to keep the position of the exercise, the higher output voltage of the low-cost system and the commercial device. According to some authors (e.g., Moritani and Mauro [23]) this behaviour and progressive increase of the signal is connected with the recruitment of new motor units and their spike amplitudes.*

**Hypothesis 3.**  *The signal from the low-cost system is reliable enough to be used in simple exercises.*

## 2. Materials and Methods

This section introduces the experimental approach carried out to test the hypotheses explained in the present work. Section 2.1 explains the steps by which the experiment was split. Section 2.2 describes the equipment used and how it was designed. Finally, Section 2.3 deals with the analysis method, where the filtration tools and assessment of the indicators are explained.

### 2.1. Experiment Scenarios

The experimental work focused on the acquisition of sEMG signals from the RF muscle of various subjects by means of two different devices. The first was a commercial device with its features indicated in Table 2, and the second device was a system built with various low-cost components, designed and deployed with a custom software interface using Matlab and Simulink [24].

The main purpose was to compare the sEMG signals of the low-cost system and the commercial device in order to be able to evaluate the feasibility of the custom system and provide a certain level of reliability.

More than 100 different cases were compared using 5 experimental subjects (3 males and 2 females) who were selected and participated in the experiment. The average participant characteristics were: age, 26 ± 2.9 years; weight, 61.8 ± 13.1 kg; height: 170 ± 6.2 cm.

**Figure 1:**
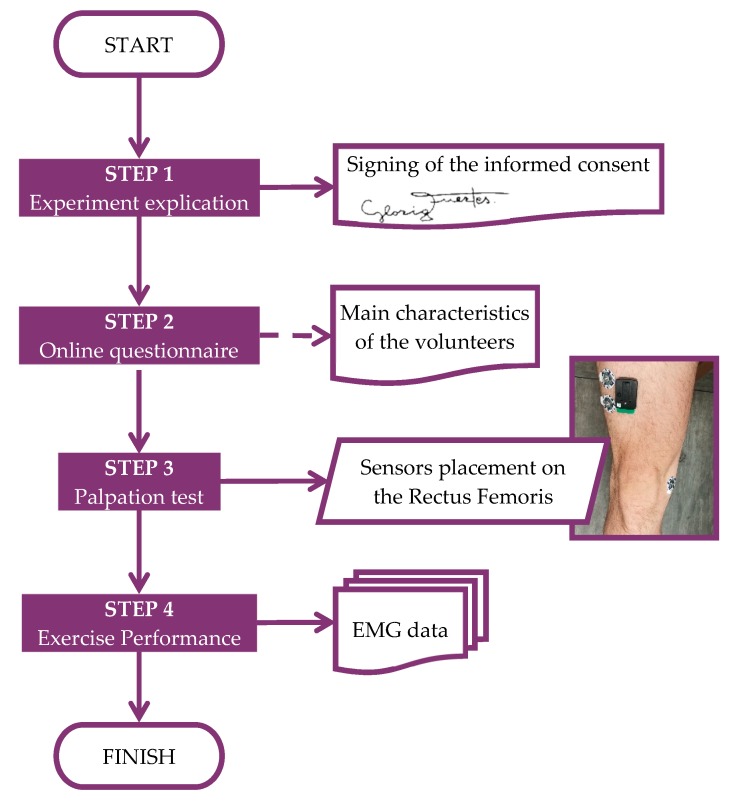
Diagram of the experiment.

Figure 1 shows the protocol and steps that each of the volunteers followed in order to complete the experiment.

Step 1: the experiment and possible risks were explained to the volunteers. Each participant had to sign an informed consent form, where details about the experiment were described to start the exercises. Each volunteer had the chance to leave the experiment whenever he or she wanted. In addition, although the informed consent form contained all the information, instructions to perform the exercise were described to each of the volunteers.

Step 2: the subject completed an electronic questionnaire. This electronic questionnaire was designed to collect certain data such as age, height or weight.

Step 3: in order to properly place the electrode sensors on the skin, a palpation test [25] was carried out. Once the RF was located, and before attaching the electrode sensors, the area was carefully cleaned, first by shaving the area with a disposable razor blade and then by cleaning it with alcohol and sterile muslin.

Step 4: execution of the exercises, as explained in Figure 1.

All the exercises carried out in the step 4 were explained, as follows. All these exercises were selected based on expert opinion, and took into account that the RF must be actively working during each exercise [26,27].

- Squat: An exercise that has demonstrated reliability in other studies and activates the muscle with an easy movement and minimal equipment [28].
- Lunge: A good exercise to strengthen the quadriceps. It is a also a basic and simple movement to do for beginners [29].
- Knee Extension: this exercise is able to be done using only the muscles of the quadriceps, making it possible to isolate the activation effect on associated muscles and focus on the selected muscle [30].
- Jump: This exercise combines the characteristics of the squat and sums movement up, therefore the analysis is more exhausting. Additionally, it is frequently used to measure the EMG signal in other studies [14,31].

The exercises were split into two categories: isometric and dynamic exercises. The isometric exercises were used when focusing on the recording of the MVC, and the dynamic exercises focused on the ability of the system to accurately register the behaviour of the wave.

In Table 2, both sets of exercises are explained. Further details of each exercise are included in the same line.

### 2.2. Equipment

The testbed used was built using two different EMG systems. The first system comprised a set of low-cost elements, and the second system was a commercial solution supported by the Mechanical Engineering Department of the University of Manchester.

Both devices were placed on the same region of the muscle to get a synchronized signal from the same muscle during exercise. The muscles selected was the RF due to its high number of motor units [33,34,35,36], something that, according to the instructions of the manufacturer of the low-cost sensor, makes better the measure of a cleaner signal with less interferences and crosstalk [37].

**Figure 2:**
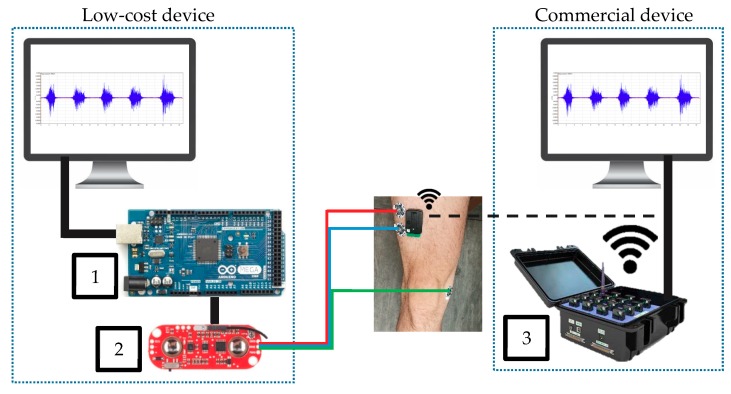
Equipment of the testbed used in the experiments. 1. Arduino Mega board. 2. Myoware EMG Muscle Sensor (SEN-13723 ROHS). 3. Delsys Trigno Wireless EMG System. A more detailed wired connection layout of the low system can be found in the supplementary material.

Figure 2 shows how the testing equipment was installed, the low-cost system and its wire-connection with the computer and with the muscle by means of three electrodes. On the right side of Figure 2 is shown the commercial system. Commercial system electrodes only need to be placed on the muscle and aligned with the muscle fibres, and its communication with the computer is by means of a wireless connection.

#### 2.2.1. Low-Cost Custom System

The low-cost system was made up of 3 components: a laptop with Matlab/Simulink [24], an Arduino board (1 in the Figure 2) and the EMG sensor (2 in the Figure 2).

The Arduino board was in charge of keeping the communication with the sensors, registering the information and then sending it to the computer, where it would be saved and later analysed. One of the main problems of the Arduino board was its limitations (power and memory), therefore the Arduino Mega was selected, because the EMG process needs a large memory to perform acquisition and filtration of the data. The main characteristics of the EMG chip sensor and the Arduino Mega board are shown in Table 3. This sensor needs three electrodes, one close to the middle of the muscle body (red line in Figure 2), the second electrode lined up with the direction of the fibre’s muscle and close to the end of it (blue line in Figure 2), and the last one placed near to a bony area as a reference (green line in Figure 2) to deal with the crosstalk signal from other muscles [37].

To place correctly the sensor pads on the skin it was necessary to locate the muscle and the direction of the muscle fibres with the help of a palpation test based on the instructions of the SENIAM (Surface ElectroMyoGraphy for the Non-Invasive Assessment of Muscles) [38]. After that, the skin should be cleaned of dirt and made hairless. A disposable razor was used to remove the hair, and alcohol with a sterile gauze was applied to clean the area. Once this step was done, the three electrode pads were attached.

#### 2.2.2. Commercial System

The second system used was a commercial one (Figure 2). Its features are summarised in Table 4. This equipment is used by a wide range of studies [39,40,41,42] because of its high accuracy and the powerful software.

In contrast to the low-cost system, this equipment is wireless, which makes it easier to install on the muscle and connect with the computer. Instead of 3 pads in different areas, this device only uses one sensor that should be lined up with the direction of the muscle fibres [43].

### 2.3. Analysis Method

Once all the exercises had been done and signals from both devices (commercial and low-cost) were saved, we began with the data analysis as follows.

On one side and from a medical point of view, an EMG signal altered by noise might cause a wrong diagnosis of the behaviour of the muscle. On the other side and from a scientific and technical point of view, a signal affected by noise does not guarantee a good interpretation of the results, reducing the reliability of the results obtained in research [44,45,46]. Therefore, due to the characteristics of the signal, it is recommended [47] to apply some kind of filter to remove the noise of the signal before starting the analysis.

The analysis method used was based on the combination of the methodology used by Dufour et al. [48] and the methodology used by Heywood et al. [14], and its stages are as follows:

1. Signal processing (signal synchronization, signal trimming and signal filtering): two independent systems were used to measure the signal, therefore it was necessary to synchronize both signals by means of a trigger. The signal was trimmed to the time of the exercise and finally filtered to denoise it. All the filtering processes were performed by EMGworks [49].
2. MVC computation and normalization of the dynamic signal: MVC was computed for every exercise. It was used to normalize the signal because each system amplifies the output signal based on its configuration (see also Isometric MVC Assessment).
3. Indicators assessment: the last stage was the assessment of the indicators in order to compare both signals (see Validation Indicators). The analysis of the signal was made by Matlab [24] and Microsoft Excel [50].

#### 2.3.1. Signal Processing

This section has been split into three parts. First it is explained how the filtering was carried out. Then it comes a short explanation about synchronizing and trimming of the signal. Finally, it is explained the assessing of the isometric MVC.

1. Signal filtering

**Figure 3:**
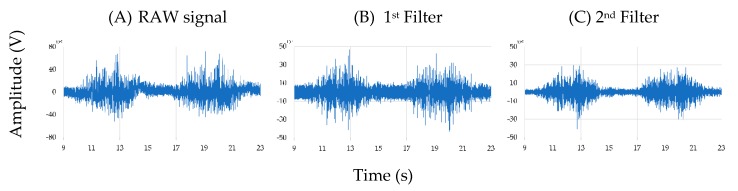
An example of the reduction of signal noise using several filters. On the left (**A**) the RAW signal between second 9 and 23. In the middle (**B**) the signal once the first filter was applied. On the right (**C**) the complete filtered signal once the second filter was applied.

As explained earlier, two different EMG devices with different technical specifications were used in the experiment. In order to denoise both signals, it was necessary to perform different signal filtering for each one. On one hand the signal from the low-cost system was filtered in 2 steps. Each filter was designed based on the evaluation of the fast Fourier transform and previous studies [4,14]. First a band pass Butterworth 40–100 Hz, order 4, was used to remove the main noise from the signal (Figure 3B). Following the low-cost system addressed some noise centred around 50 Hz, that was filtered by a stop band Butterworth 45–55 Hz, order 4 (Figure 3C).

On the other hand, the signal from the commercial system was filtered by a band pass Butterworth 40–400 Hz, order 4. The data processing algorithm was found to be appropriate by many researchers such as Wei et al. [51].

2. Signal synchronization and trimming

**Figure 4:**
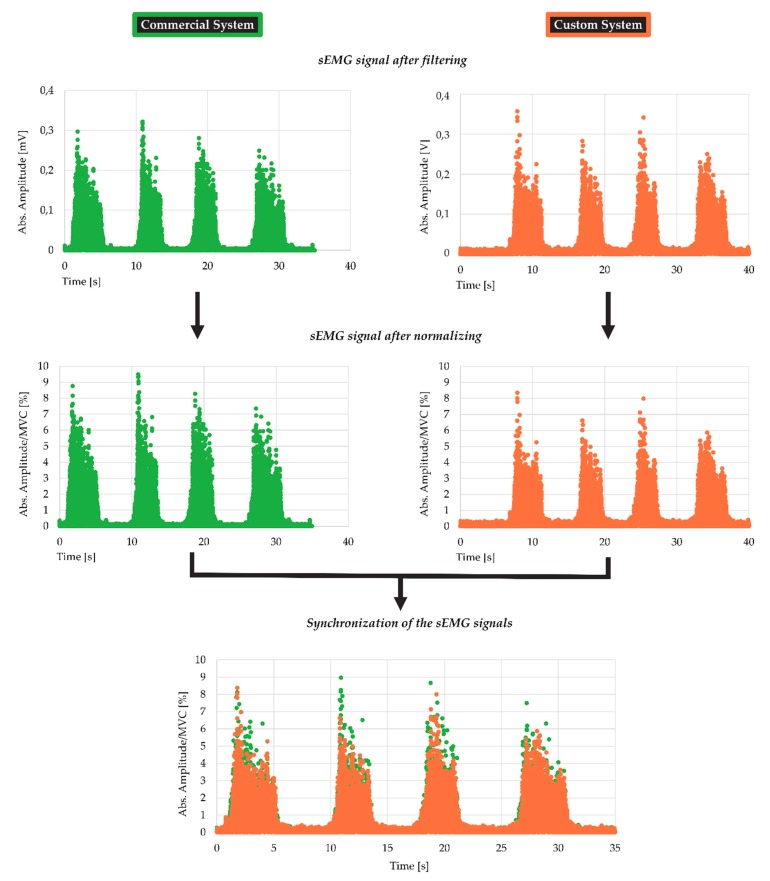
Signal treatment procedure. On the top the absolute value of the signal after filtering is plotted (Section 2.3.1). In the middle, signal of both systems (commercial and low-cost custom) once they were normalized by means of the MVC. On the bottom of the figure both signals are overlapped and trimmed according to the duration of the exercise. On the right (orange) signal from the low-cost custom system and on the left (green), signal from the commercial system.

Before starting the exercises, volunteers were asked to knock the floor with their sensorized leg. In this manner, a single precise indication would be generated to be able to overlap and synchronize both signals, the signal from commercial equipment and the signal from the low-cost system. When both signals were synchronized, it was necessary to determine the duration of each of the exercises and trim both signals. This technique was semi-automatic by the use of a custom-designed script in Matlab [24]. It is important to note that the sEMG signal was changed with a single polarity with the purpose of ensuring an average not equal to zero (Figure 4).

3. MVC assessment

Once the signals of both sets of equipment were filtered, synchronized and trimmed, the next step was assessing the isometric MVC of each exercise and then normalizing the signal to enable calculation of the indicators that check the viability of the low-cost system.

#### 2.3.2. Isometric MVC Assessment

The maximal contraction of the quadriceps of the volunteers was achieved through three different exercises as listed in Table 2. Each one of these exercises was used to normalize the related dynamic EMG signal. The output ranges of each set of equipment are quite different, making signal normalization necessary.

**Figure 5:**
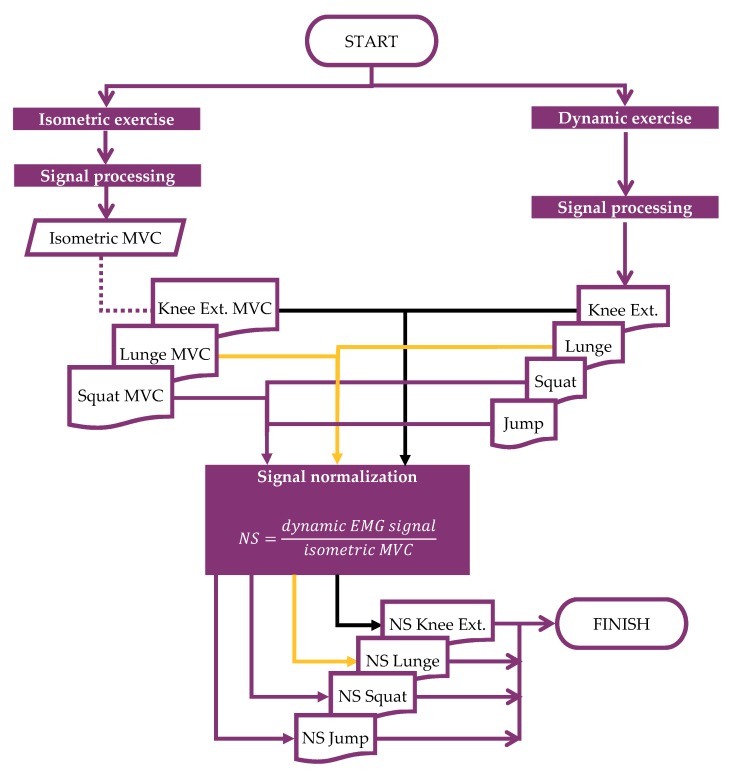
Diagram followed to normalize the sEMG signal from the dynamic exercises.

The diagram in Figure 5 shows the steps that have been followed for the normalization of the signal. On the left side of the diagram is shown the evolution of sEMG signal of the isometric exercises (knee extension, lunge and squat). Those signals were used to obtain the MVC from the waveform of each exercise and from the commercial and low-cost system separately. On the right side can be seen the treatment of the sEMG signal of the dynamic exercises, from the acquisition to the signal processing, where it was denoised. After that the dynamic sEMG signal was normalized according to the formula indicated in the same figure (Figure 5) by means of the corresponding MVC value.

#### 2.3.3. Validation Indicators

In order to achieve a successful validation of the low-cost signal, a set of validation indicators were calculated. Each indicator was selected based on a different criterion, explained in the following lines.

1. Spearman’s correlation: assesses the monotonic relationship of two variables. It can move between −1 and 1, 1 when signals are identical and −1 when signals are fully opposed. This correlation has been used in several studies [52,53] to compare signals from different sources with good results.
2. Energy ratio: defined as the energy (E) as in Equation (1) [54], the energy ratio moves between 0 (no similarity) to 1 (fully identical), and it compares the amount of energy step by step, testing if the sEMG waves have the same form in both scenarios.
  (1) $$E=\int_{0}^{t}{\left|x\left(t\right)\right|}^{2}dt$$
3. Linear correlation coefficient (LCC): the LCC has been calculated according to Equation (2); x represents the values of the low-cost system and y the values of the commercial system. Linear correlation is bounded between −1 and 1. When LCC > 0 it is a positive correlation. In case LCC < 0 it is a negative correlation. If LCC = 0 there is no correlation between both variables, and is an uncorrelated relation. One example where this correlation has been used is in the work undertaken by Andrea et al. [31].
  (2) $$LCC=\frac{\sum \left(x-\bar{x}\right)\left(y-\bar{y}\right)}{\sqrt{\sum {\left(x-\bar{x}\right)}^{2}\sum {\left(y-\bar{y}\right)}^{2}}}$$
4. Cross-correlation coefficient (CCC): One widespread method (3) to compare EMG signals [55,56]. The CCC can stay between −1 to 1, where −1 means negative correlation and 1 positive correlation.
  (3) $$CCC=\frac{n(\sum xy)-\left(\sum x\right)\left(\sum y\right)}{\sqrt{\left[n\left(\sum x^{2}\right)-{\left(\sum x\right)}^{2}\right]\left[n\left(\sum y^{2}\right)-{\left(\sum y\right)}^{2}\right]}}$$

### 2.4. Volunteers Classification

The validation of the low-cost system was carried out using volunteers who participated in the experiment. There were three male and two female volunteers, with an average age of 26.6 ± 2.9 years, an average height of 170 ± 6.2 cm and an average weight of 61.8 ± 13.1 kg.

All the volunteers were informed about the experiment and agreed to use all the data for scientific purposes. In addition, they were informed that the experiment could be stopped at any time as they wished, with no obligation to complete it.

### 2.5. Validation Analysis

The dataset involved more than 100 different curves to carry out the comparative and validation work, because each volunteer repeated the exercises a number of times. Results are shown below.

Validation analysis was split into two sections. The first section compares the isometric sEMG output signal of the commercial and low-cost devices. After that results of the indicators (Section 2.3.3) are explained.

## 3. Results

The following section presents the results obtained in this work. First it describes the sample of the volunteers that participated in the experiment. After that it presents the results of the experiment in two different stages.

### 3.1. Isometric Exercises

As is known, muscle work activation level and the output signal are dependant according to previous studies [36,57]. Therefore, the main aspect of this section is to show the MVC values, gathered from the isometric exercises, in order to check if the low-cost system behaves as the commercial system as explained before and confirms the reliability of the system.

**Figure 6:**
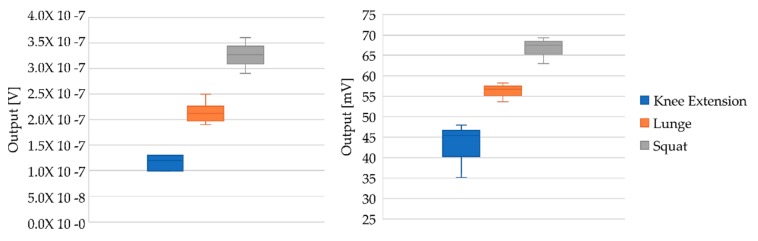
MVC distribution according to the exercise and equipment used.

As can be seen in the chart in Figure 6, MVC values of all the volunteers were represented. They have been grouped by exercise type and sorted by commercial or custom device.

### 3.2. Indicators Assessment

Results of the comparison between the commercial and low-cost system based on the indicators can be found below. These indicators provide information about the signal and its reliability when this system is used for simple exercises, as in the experiment.

Table 5 shows a summary of the indicators. Parameters include the maximum (max), minimum (min), mean (μ) and standard deviation (σ) for a better understanding and to compare better both signals.

For each factor (SC, E_CS_/E_CMS_, LCC and CCC) the maximum, minimum, mean and standard deviation are shown.

## 4. Discussion and Conclusions

This article presented our study validating the low-cost sEMG system. The system was developed using low-cost components and Matlab/Simulink software with the main idea of being an economic alternative to commercial systems, keeping in mind the cutting-edge limitations of the low-cost technology.

To reach that objective, an experiment to compare the output sEMG signal between a commercial system and the proposed low-cost system was carried out. In that experiment each volunteer was sensorized with both systems to register the sEMG signal of the rectus femoris (RF) while performing some isometric and dynamic exercises. After that sEMG signal was used to carry out an analysis following the explanation presented in Section 2.3 to validate the low-cost device. First, the signal was synchronized, trimmed and denoised. Secondly, the MVC (maximal voluntary contraction) was assessed to normalize the signal and finally some indicators to compare the similarity of both signals were assessed.

Throughout the study some hypothesises were taken into account. The first one is related to the relationship between the output sEMG signal and the exercise, making it possible to detect the exercise with the output signal values. The second hypothesis states that the more difficulty to perform one exercise, the higher output voltage is registered. Finally, the third hypothesis enclosed the possible uses of the low-cost system for simple exercises, up to date.

The sEMG signal acquisition was done, as can be seen in Figure 2, by two different and isolated computers. Because of that, both signals must be synchronized after the exercises. To that aim before starting any exercise, each volunteer activated a trigger as matching point between both signals (Figure 5). More information related to that can be found in the Section 2.3.1.

The exercises each volunteer had to perform were split into two groups, the isometric exercises and dynamic exercises. The isometric exercises were used to obtain the MVC of each exercise to compare the behaviour of the signal based on the muscle activation level. The dynamic exercises were used to assess the four indicators to compare the sEMG signal from the low-cost and the commercial devices.

One of the first stages for the analysis was the signal denoising. It is important to highlight that although the muscle is not stressed there could be still a low noise after being filtered. One of the reasons why this may happen is due to the way the system is set up and the inherent noise of the hardware employed [12]. Connections were done with braided cable, although they could be improved in the future by means of welding terminals instead the pin system of the Arduino Mega board. In addition, for a better reduction of the noise, it might be interesting to isolate the system, although it is very difficult to remove the noise in an efficient way [13]. All those characteristics must be studied and improved in future upgrades.

Results of the isometric exercises are plotted in Figure 6, on the right side the results from the custom system and on the left side the results of the commercial system. As can be seen, in both cases the tendency is the same, the output voltage of the signal increases according to the difficulty to maintain the isometric position (second hypothesis). In addition, the output values for each exercise define an average value, which makes it possible to identify the exercise, thereby fulfilling the first of the hypotheses proposed. Aside from this, all the similarities suggest that the low-cost device works in a satisfactory way, so it is possible to continue with the validation of the device (assessment of the indicators). In addition, it is important to note that this research confirms the results related to muscular behaviour in accordance with the subject studied [4].

The next stage of the study was the assessment of the indicators listed in the Section 2.3.3. They were chosen to estimate the similarity between the two signals in a numerical way, so that the results could validate the low-cost system. The Spearman’s coefficient calculated in all the exercises is above 0, which means that the correlation between both signals is positive. In addition, results fluctuate between a minimum of 0.51 and a maximum of 0.96, with an average above 0.60, which indicates similarity between both signals. The next indicator was the energy ratio, the amount of energy along the exercise of the low-cost device divided by the amount of energy along the exercise of the commercial system. This indicator varies from 0 to 1, where 0 is no similarities and 1 is 100% similarity between both signals. Results from the experiment are from a minimum of 0.71 to a maximum of 0.98, with an average above 0.80. Those discrepancies are mainly due to the noise recorded by the custom equipment, since the signal shape of the custom and commercial system is very similar, as can be seen in Figure 4. The third factor is the linear correlation coefficient. It also varies between 0 and 1 (0, no correlation; and 1, perfect correlation). In this case, the maximum, minimum and average values are very close to 1 (maximum 1, minimum 0.98 and average 0.99), therefore linear correlation is almost 100%. The last indicator is the cross-correlation coefficient (CCC). It moves between −1 to −1, where −1 means negative correlation and 1 positive correlation. Results show a mean around 0.65, with a maximum of 0.87 that means a good correlation between the signals.

All these findings indicate that there is a excellent agreement between the custom and the commercial system. As a result, the third hypothesis is valid. The objective of this article has been successfully achieved: Build a user-friendly system with low-cost parts.

In any case, although the achieved results were satisfactory, it is important to be aware of some limitations that were found in the use of this low-cost system.

The first limitation, as mentioned above, is the problem with noise from both the assembly and the equipment.

A second issue is related to the delay of the signal. The low-cost system has a slightly higher delay than the commercial system, which could be a problem when tracking the signal in vivo. This delay is lower than a second and must be studied in future analysis.

Also, it is important to have in mind that sEMG signal is very sensitive to the cleanliness of the surface of positioning of the sensors.

One more factor is the sampling frequency, since the system is weighed down by the Arduino board.

Due to the fact that the system can be improved in future studies, more analysis and accurate study of the limitations are going to be studied in the future.

## Figures and Tables

**Table 1:** Price comparison between commercial systems and the low-cost electromyography (EMG) system.

Equipment		Prize (€)
Commercial Device	Delsys Trigno	(around) 20,000
	Cometa	(around) 15,000
Low-Cost Sensors	Bitalino	Up to 150
	Myoware EMG + Arduino Mega	100

**Table 2:** Description of the isometric contraction exercises (top), and description of the dynamic exercises (bottom). Images created by Miguel Gómez Palacios [32].

**Isometric Contraction**									
**Exercise**	* N° rep	**Duration (Seconds)**	**Instructions**				**Description**		
Squat	3	30	Contraction of 90° knee flexion				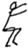		
Lunge	3	30	Contraction of the forward leg to 90° knee flexion				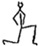		
Knee extension	3	30	Seat on a chair, 180° knee extension and leg in horizontal position				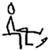		
**Dynamic exercise**									
**Exercise**	* N° rep	** reps	**Instructions**	**Description**					
Squat	3	1/10	3 slow speed down and slow speed up	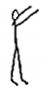	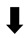		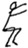		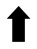
Lunge	3	1/10	3 slow speed down and slow speed up	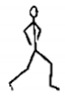	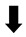		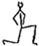		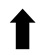
Knee extension	3	1/10	3 slow speed down and slow speed up	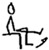			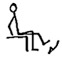		
Jump	3	2/10	3 slow down to 90° knee flexion and jump as high as possible. Once landed slow down to 90° knee flexion and return to stand up position	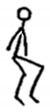	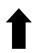	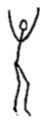	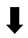	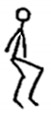	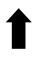

* N° rep: number of repetitions; ** reps: repetitions per second.

**Table 3:** Technical specifications of the Arduino Mega board and the low-cost EMG chip.

Arduino Mega	
Microcontroller	ATmega2560
V_in_ (V)	7–12
V_out_ (V)	6–20
Digital Inputs/Outputs	54
Analogue Inputs	16
Flash Memory (Kb)	256
SRAM (Kb)	8
EEPROM (Kb)	4
Clock Speed (MHz)	16
**sEMG Sensor**	
Supply (V)	2.9–5.7
Output modes	EMG Envelope/Raw EMG
Size (cm)	2.08 × 5.23

**Table 4:** Delsys Trigno Wireless EMG system. Technical information.

**EMG**	
Dimensions (mm)	27 × 37 × 13
Mass (g)	14
EMG Signal Input Range (mV)	11/22
EMG Signal Bandwidth (Hz)	20–450/10–850
EMG Contact Dimensions (mm)	5 × 1
Contact Material	99.99% silver
**Accelerometer**	
Accelerometer Bandwidth (Hz)	24–470
Accelerometer Range (g)	±2, ±4, ±8, ±16
**Gyroscope**	
Gyroscope Bandwidth (Hz)	24–360
Gyroscope Range (dps)	±250, ±500, ±1000, ±2000
**Magnetometer**	
Magnetometer Bandwidth (Hz)	50
Magnetometer Range (μT)	±4900

**Table 5:** Current validity. SC, Spearman’s correlation; E_CS_, energy low-cost system, E_CMS;_ energy commercial system; LCC, linear correlation coefficient; CCC, cross-correlation coefficient.

		Exercise			
		Lunge	Knee Extension	Squat	Jump
SC	max	0.96	0.71	0.64	0.70
	min	0.51	0.53	0.57	0.59
	μ	0.67	0.63	0.61	0.6
	σ	0.2	0.08	0.03	0.1
E_CS_/E_CMS_	max	0.92	0.82	0.91	0.80
	min	0.71	0.98	0.82	0.85
	μ	0.81	0.88	0.92	0.8
	σ	0.11	0.11	0.11	0.6
LCC	max	0.99	1	1	0.97
	min	0.97	0.97	0.98	0.93
	μ	0.99	0.99	0.99	0.95
	σ	0.01	0.01	0.01	0.02
CCC	max	0.87	0.83	0.72	0.71
	min	0.51	0.5	0.52	0.5
	μ	0.68	0.64	0.62	0.61
	σ	0.15	0.16	0.08	0.04

## Supplementary Materials

The following are available online at https://www.mdpi.com/1424-8220/19/23/5214/s1.
