# Supplementary figures and images for: Validation of a Low-Cost Electromyography (EMG) System via a Commercial and Accurate EMG Device: Pilot Study
Source: Sensors (Basel). 2019 Nov 28;19(23):5214. doi: 10.3390/s19235214 (PMC6928739; doi:10.3390/s19235214)

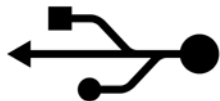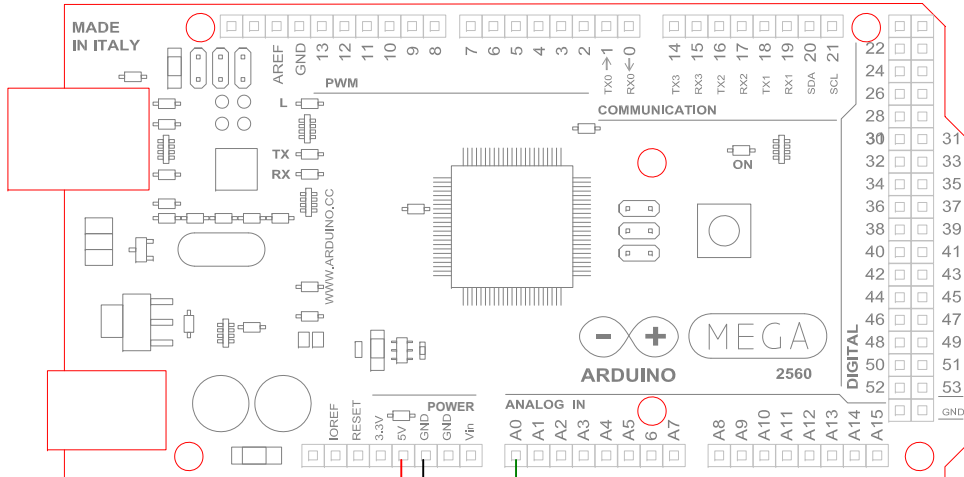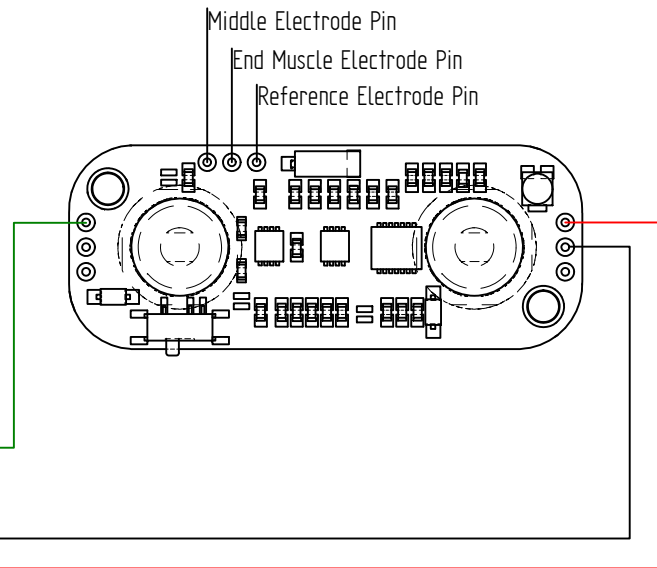

Supplement: Supplementary file 1 [file sensors-19-05214-s001.pdf]
